# The Nootropic Drug A-Glyceryl-Phosphoryl-Ethanolamine Exerts Neuroprotective Effects in Human Hippocampal Cells

**DOI:** 10.3390/ijms21030941

**Published:** 2020-01-31

**Authors:** Simona Daniele, Giorgina Mangano, Lucia Durando, Lorella Ragni, Claudia Martini

**Affiliations:** 1 Department of Pharmacy, University of Pisa, via Bonanno Pisano 6, 56126 Pisa, Italy; simona.daniele@unipi.it; 2 Angelini RR&D (Regulatory, Research & Development)—Angelini S.p.A., Piazzale della stazione snc, S. Palomba-Pomezia, 00071 Rome, Italy; giorgina.mangano@angelinipharma.com (G.M.); lucia.durando@angelinipharma.com (L.D.); lorella.ragni@angelinipharma.com (L.R.)

**Keywords:** α-glycerylphosphorylethanolamine, phospholipid precursor, aging, cellular membrane

## Abstract

Brain aging involves changes in the lipid membrane composition that lead to a decrease in membrane excitability and neurotransmitter release. These membrane modifications have been identified as contributing factors in age-related memory decline. In this sense, precursors of phospholipids (PLs) can restore the physiological composition of cellular membranes and produce valuable therapeutic effects in brain aging. Among promising drugs, alpha-glycerylphosphorylethanolamine (GPE) has demonstrated protective effects in amyloid-injured astrocytes and in an aging model of human neural stem cells. However, the compound properties on mature neuronal cells remain unexplored. Herein, GPE was tested in human hippocampal neurons, which are involved in learning and memory, and characterized by a functional cholinergic transmission, thus representing a valuable cellular model to explore the beneficial properties of GPE. GPE induced the release of the main membrane phospholipids and of the acetylcholine neurotransmitter. Moreover, the compound reduced lipid peroxidation and enhanced membrane fluidity of human brain cells. GPE counteracted the DNA damage and viability decrease observed in in vitro aged neurons. Among GPE treatment effects, the autophagy was found positively upregulated. Overall, these results confirm the beneficial effects of GPE treatment and suggest the compound as a promising drug to preserve hippocampal neurons and virtually memory performances.

## 1. Introduction

Brain aging has been related to peculiar structural and functional alterations that include neuronal and astrocyte degeneration, neuroinflammation and changes in the plasma membrane composition [1,2,3]. Specifically, brain cells undergo a decline of polyunsaturated n-3 fatty acids (3-PUFA) and docosahexaenoic acid (DHA) levels within aging. Such alterations lead to a decrease in membrane fluidity and cholinergic activities, which depend on phosphatidylcholine (PC) and PUFAs for excitability and neurotransmitter release, via retarded Na^+^/Ca^+^ channels [4,5]. Memory decline and the reduced learning abilities in the elderly are supposed to be the consequence of a decreased quantity of phospholipids (PLs) and/or PUFAs in the brain. Not surprisingly, the changes in the lipid composition of brain cells have been correlated with neurodegenerative disease onset [6,7] and the presence of unsaturated membranes has been linked to shorter life span in different animal species [8,9,10]. Since PLs can promote PUFA transport, external PLs can restore the physiological composition of brain cellular membranes and produce valuable therapeutic effects in brain aging [4,5,11,12]. In this respect, explicit memory has been demonstrated to be enhanced by phosphatidylcholine (PC) administration via the release of choline [1,13] which is a constituent of the cellular membrane and an acetylcholine (Ach) precursor [1]. In particular, the Ach precursor, choline alfoscerate, and the PC biosynthesis intermediary, citicoline, have been proven to enhance memory and cognitive function in humans and even in Alzheimer’s patients [14]. Finally, lifespan of yeast and human-derived cell cultures has been prolonged by phosphatidylethanolamine (PE) and its precursor ethanolamine [1,15]. Among biological precursor membrane constituents, alpha-glycerylphosphorylethanolamine (GPE) has been emerging as a promising PE and PC donor with the ability to promote Ach synthesis [1,16,17]. GPE has been proven to possess protective effects in amyloid-injured astrocytes [16] and in an aging model of human neural stem cells [1]. However, the compound properties on neuronal cells remain unexplored. The aim of the present study was to investigate GPE effects on human hippocampal neurons. These cells have been widely demonstrated to have functional cholinergic transmission [18] and play a pivotal role in cognition, learning and memory [19], thus representing a valuable cellular model to explore GPE neuroprotective properties.

## 2. Results

### 2.1. GPE Induced the Release of Phospholipids and Acetylcholine in Human Hippocampal Neurons

**Figure 1:**
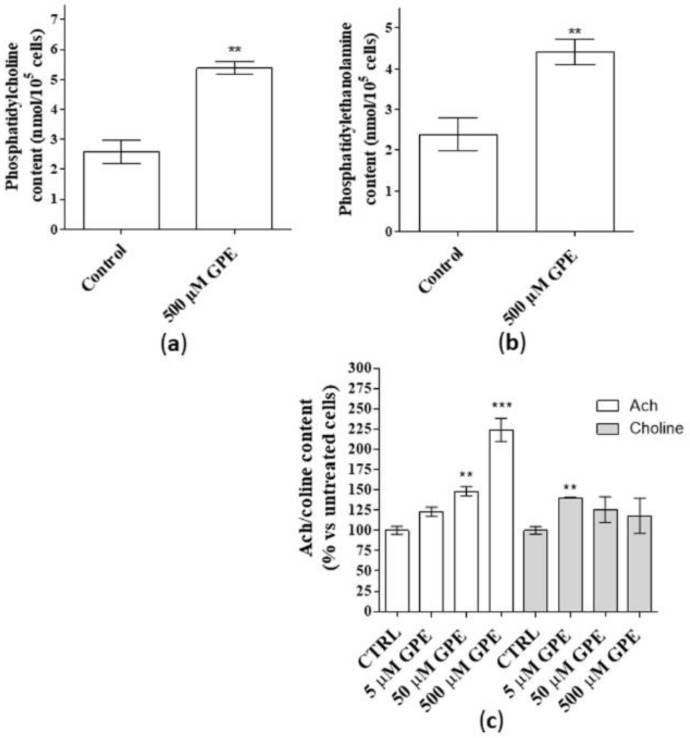
(**a**–**c**) Human hippocampal neurons were incubated with saline buffer (control cells) or α-glycerylphosphorylethanolamine (GPE) at the indicated concentrations for seven days. Following incubation, the contents of phosphatidylcholine (PC, panel a), phosphatidylethanolamine (PE, panel b), Ach/choline (panel c) were determined by fluorometric methods as described in the Methods section. (**a**,**b**) Data are the mean ± SEM of three different experiments, each performed in duplicate expressed as nanomol PC/10^5^ cell sample (**a**) and nanomol PE/10^5^ cell sample (**b**). Statistical analysis was performed by unpaired t-test with Welch’s correction: ** *p* < 0.01 versus control. (**c**). Data are shown as pmol/well of Ach (white bars) or choline (grey bars), expressed as percentage amount with respect to untreated cells, and they are the mean ± SEM of two different experiments, each performed in duplicate. Statistical analysis was performed by one-way analysis of variance (ANOVA) with Bonferroni’s corrected t-tests for post-hoc pair-wise comparisons: ** *p* < 0.01, *** *p* < 0.001 versus control.

As a first step, the ability of GPE to produce phospholipids and Ach was investigated. Human mature hippocampal neurons were used in the present work to test the effects of GPE treatment. In this cellular model, GPE did not affect cell number following seven days of treatment (data not shown), confirming the lack of toxicity of the compound at the tested concentrations (5–500 µM), [1,16]. Challenging human hippocampal neurons with GPE for seven days induced a significant release of PC and PE (Figure 1, panels a and b). These data are consistent with our previous results obtained in human NSCs [1]. Furthermore, GPE enriched significantly Ach content in a concentration dependent manner with a maximal effect at 500 µM (Figure 1c, white bars). In contrast, the release of choline was significant only for low GPE concentrations (Figure 1c, grey bars) and not concentration-dependent (Figure 1c, grey bars).

### 2.2. GPE Favoured the Proper Membrane Function

**Figure 2:**
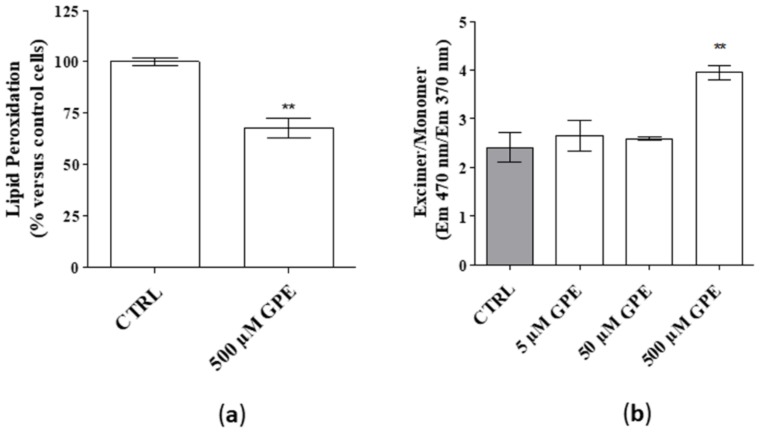
(**a**,**b**) Human hippocampal neurons were incubated with saline buffer (control cells) or GPE at the indicated concentrations for seven days. (**a**) Following incubation, cells were collected and lysed, and lipid peroxidation was estimated by a fluorometric assay, as described in the Methods section. Data are expressed as percentage amount with respect to untreated cells and they are the mean ± SEM of three different experiments, each performed in duplicate. Statistical analysis was performed by unpaired *t*-test with Welch’s correction: ** *p* < 0.01 versus control. (**b**) Human hippocampal neurons were treated as in (**a**). Following incubation, membrane fluidity was attained by measuring the ratio of pyrene monomer (EM max. 370 nm) to excimer (EM 470 nm) fluorescence. Data are the mean ± SEM of two experiments, each performed in duplicate. Statistical analysis was performed by one-way analysis of variance (ANOVA) with Bonferroni’s corrected *t*-tests for post-hoc pair-wise comparisons: ** *p* < 0.01 versus control.

Neuronal membranes are rich in polyunsaturated fatty acids, which are particularly susceptible to oxidative stress, leading to identify products of lipid peroxidation as biomarkers of neurological disorders [20]. In this context, the effect of the PL precursor on the level of lipid peroxidation was measured. As depicted in Figure 2 (panel a), GPE significantly reduced the percentage of lipid peroxidation. Furthermore, challenging hippocampal cells with GPE (500 µM) significantly improved membrane fluidity (Figure 2b), which is pivotal for proper membrane function and cell viability [7]. Globally, these data demonstrate that GPE improves membranes’ quality of human hippocampal neurons.

### 2.3. GPE Induced Autophagy in Human Hippocampal Neurons

**Figure 3:**
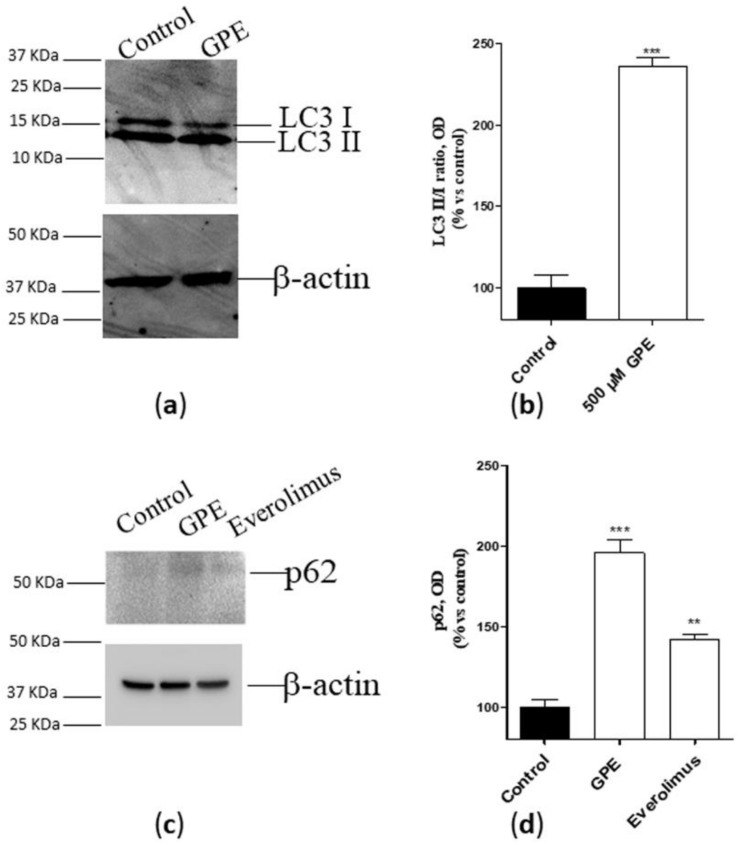
(**a**,**b**) Human hippocampal neurons were incubated with saline buffer (control cells) or GPE 500 µM for seven days. Following incubation, cells were collected and lysed. The expression of the autophagic marker LC3 (I and II) was detected by Western blotting analysis. (**c**,**d**) Human hippocampal neurons were incubated with saline buffer (control cells) or GPE (500 µM) or everolimus (50 nM) for seven days. Following incubation, cells were collected and lysed. The expression of the autophagic marker p62 was detected by Western blotting analysis. (**a**,**c**) A representative Western blotting is shown. (**b**,**d**) Optical density was measured by Image J software. The data are reported as percentage of control cells, and they are the mean ± SEM of two different experiments, each performed in duplicate. Statistical analysis was performed by unpaired t-test with Welch’s correction: ** *p* < 0.01, *** *p* < 0.001 versus control.

Autophagy is considered as a crucial homeostatic mechanism in healthy cells and a cytoprotective response in aging- and disease-related metabolic challenge [20]. Considering the pivotal role of PLs in autophagosome formation and fusion [21], we next verified whether GPE can affect the autophagic process in human hippocampal neurons. As depicted in Figure 3 (panel a and b), the PL precursor strongly enhanced the conversion of LC3 I to LC3II, as demonstrated by the increase of the LC3 II/LC3 I ratio. To confirm these data, a western blot analysis of the autophagic protein p62 [22] was performed, using the mTOR inhibitor [23,24] everolimus as a positive control. GPE was proven to significantly augment p62 accumulation, even to a greater extent than everolimus (Figure 3c,d). These data demonstrate that GPE favors autophagy in human hippocampal neurons.

### 2.4. GPE Showed Cytoprotective Effects in a Model of Aged Neurons

As a final step, the putative cytoprotective properties of the compound were tested in a model of aged neurons. To mimic cellular aging, the hippocampal cells were maintained for 21 days in vitro (DIV) [25].

**Figure 4:**
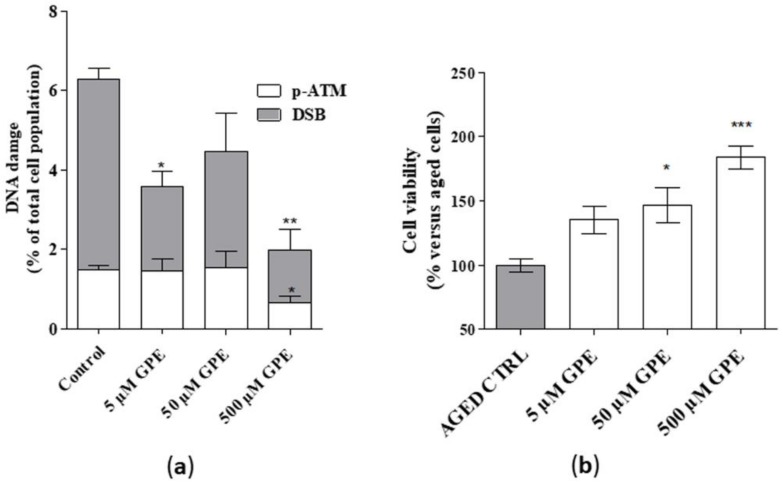
(**a**) Human hippocampal neurons were incubated with saline buffer (control cells) or GPE (5, 50, and 500 µM) for 21 days in vitro (DIV). Following incubation, cells were collected and lysed. DNA damage (DSB and phospho-ATM) was quantified by cytofluorimetric analysis (Muse^TM^). Data are the mean ± SEM of two different experiments, each performed in duplicate, and are reported as percentage of total cell population. (**b**) Human hippocampal neurons were treated as in A. Following incubation, cellular viability was measured by MTS assay. Data are the mean ± SEM of two different experiments, each performed in duplicate, and reported as percentage of untreated aged cells (control). Statistical analysis was performed by one-way analysis of variance (ANOVA) with Bonferroni’s corrected t-tests for post-hoc pair-wise comparisons: * *p* < 0.05, ** *p* < 0.01, *** *p* < 0.001 versus control (aged cells).

Detectable levels of common markers of DNA damage, i.e., DSB (Double Strand break) and phosphor-ATM (Figure 4a), were found in aged hippocampal cells, thus confirming the validity of the cellular model. GPE, tested at 5 and 500 µM, reduced significantly the percentage of cells presenting DSB (Figure 4a). Furthermore, a significant reduction in the level of phosphor-ATM was noticed upon incubation with GPE (Figure 4a). Moreover, a significant enhancement in cell viability in the presence of GPE was evidenced (Figure 4b). Interestingly, these effects appeared to be concentration-dependent. Globally these data demonstrate that the tested compound can reduce DNA damage in human hippocampal neurons and possess cytoprotective effects.

## 3. Discussion

In this paper, GPE potential neuroprotective properties were investigated in adult human hippocampal neurons. In particular, GPE was demonstrated to induce the release of the main membrane phospholipids, PC and PE, and of the neurotransmitter Ach. Moreover, challenging hippocampal cells with GPE reduced lipid peroxidation and enhanced membrane fluidity. GPE protected 21 DIV-aged neurons from DNA damage and viability decrease. Among GPE treatment effects, the induction of autophagy was demonstrated.

Brain aging involves a slow impairment of memory function, which has been related to an impairment of brain cell functions. One contributing factor to this memory decline has been identified in a peculiar change of neuronal lipid composition [4]. In particular, reduced levels of PC and/or PUFAs in brain cells have been associated to a decrease in membrane excitability and neurotransmitter release [4,5] that lead to insufficient synaptic transmission and finally to memory impairment. Consistently, the administration of drugs able to restore membrane composition and functionality can ameliorate memory performances [14]. Among these compounds, GPE has emerged in preserving the well-being of astrocytes and neural stem cells [1,4,15,16,17]. Herein, the effects of GPE on membrane and cellular well-being were verified in adult human hippocampal cells. These cells have been widely demonstrated to present functional cholinergic transmission [18] and play a pivotal role in cognition, learning and memory [19], and thus representing a valuable cellular model to explore nootropic properties of drugs.

When hippocampal neurons were incubated with GPE, a significant release of PC and PE was evidenced, as previously demonstrated in human NSCs [1]. Furthermore, GPE significantly enriched Ach content, in a concentration dependent manner, supporting the nootropic properties of the compound. To note, improved learning and memory has been widely associated with an enhancement in cholinergic neurotransmission in the hippocampus [14,26,27,28]. Our data suggested an augmentation of Ach that could be beneficial for memory consolidation in neurons.

Other compounds have been demonstrated to increase Ach levels in neurons that can contribute to the enhancement of memory and hippocampal plasticity via interfaces with non-neuronal cells [29]. These findings may account for the beneficial effects of GPE that have been proven in astrocytes and NSCs too [1,16]. By contrast, choline release was concentration-independent and significant only for low GPE concentrations. These results could be explained considering that the measured choline release following seven day-treatment is affected by the simultaneous requisition of PC in the Ach synthesis pathway [30].

Furthermore, GPE was proven to significantly reduce the percentage of lipid peroxidation. This main oxidative mechanism is responsible for the creation of highly reactive derivatives, often damaging other membrane components, proteins, and nucleic acids [9,31]. Consistent with our data, lipid peroxidation has been significantly modified by PL supplementation, such as DHA/eicosapentaenoic acid (EPA), in streptozotocin-treated rats [32]. Challenging hippocampal cells with GPE significantly improved membrane fluidity, which is pivotal for proper membrane function and cell viability [7]. Indeed, membrane fluidity modulates the potency of ligand binding to membrane receptors and the activity of membrane enzymes, receptors, channels, and transporters [7,33] and its modifications have been associated to age-related pathologies, including Alzheimer’s disease [34,35,36]. In this respect, and consistent with our data, DHA-phospholipid supplementation has been demonstrated to increase erythrocyte membrane fluidity in elderly patients with mild cognitive impairment [37].

To get insight into the putative intracellular mechanisms supporting GPE-elicited effects, we investigated the induction of autophagy in neuronal cells. The results showed that GPE significantly favored the autophagic process. Consistently, PLs essentially contribute to autophagosome formation and fusion [21]. These results are of pivotal significance since autophagy has been suggested as a protective mechanism in neurons undergoing physiological aging [38].

As a final step, the cytoprotective properties of the compound were tested in a model of aged neurons, i.e., adult hippocampal cells maintained for 21 DIV [25]. Moderate but detectable levels of common markers of DNA damage were found in these neuronal cells, thus confirming the validity of the cellular model. Our preliminary data showed that GPE reduced significantly the percentage of cells presenting DNA damage and significantly enhanced the viability of aged neurons in a concentration-dependent manner. Of note, the protection on aging-associated DNA damage was moderate, although statistically significant, and will require further investigation to confirm the obtained results.

Globally these data demonstrate that the tested compound can reduce DNA damage in human hippocampal neurons and possess cytoprotective effects. Increasing evidences show that restoring PL content can ameliorate memory loss and cognitive deficiency [39], as recently confirmed in a mouse model of AD [40]. Furthermore, a delayed treatment with choline alfoscerate has been proven to reduce neuronal death and to increase hippocampal neurogenesis [41].

In conclusion, the beneficial effects of GPE on membrane composition and functionality of human hippocampal neurons was proven in the present paper. Furthermore, the tested compound was able to reduce DNA damage and viability decrease of the same aged cells.

Of note, adult hippocampal cells were challenged with medium-high GPE concentrations, greater than those achieved in vivo with classic oral supplementation. In this sense, it has to be underlined that different active metabolites can be generated in vivo rather than in vitro, thus reducing the dose required for the therapeutic action. Further experiments will investigate the putative generation of GPE metabolites in vitro and their functional activities.

## 4. Materials and Methods

### 4.1. Cell Culture of Hippocampal Neurons and Pharmacological Treatments

Adult human hippocampal neurons were purchased from Innoprost (B-Bridge International) and cultured as described [42]. The neuronal cells were thawed and directly seeded for the experiments.

GPE was provided by Angelini S.p.A. Stock solutions of varying GPE concentrations were created by dilution with phosphate-buffered saline (PBS). GPE was used at 5, 50, or 500 µM, on the basis of previous reports [1,16].

All other reagents were of the highest commercially available grade and obtained from standard commercial sources.

### 4.2. Quantification of Phosphatidylcholine (PC) and Phosphatidylethanolamine (PE)

Hippocampal neurons were seeded in 6-multiwell plates (1 × 10^5^ cells/well) and incubated with saline buffer (control cells) or 500 µM GPE for seven days. Following incubation, phospholipids’ content was determined by a fluorometric method. Briefly, an enzyme-coupled reaction was used to hydrolyze PC to choline, which subsequently oxidizes the OxiRed probe, resulting in development of fluorescence (Ex/Em 535 nm/587 nm, ab83377 Abcam, Cambridge, UK). Similarly, an enzyme-coupled reaction was used to hydrolyze PE to an intermediate, which converts a colorless probe to a fluorescent product via enzymatic reaction (Ex/Em 535/587, #K499-100 Biovision, Biovision Incorporated, Milpitas, CA, USA). The data are expressed as nanomole of PC or PE/sample. Samples were verified for the presence of a comparable number of cells in each well following GPE treatment.

### 4.3. Quantification of Acetylcholine (Ach) and Choline Release

Adult hippocampal neurons were seeded in 6-multiwell plates (1 × 10^5^ cells/well) and incubated with saline buffer (control cells) or 5, 50, or 500 µM GPE for seven days. Following incubation, cells were collected, and Ach and choline contents were determined by a fluorometric method. In brief, free choline is oxidized to betaine, via the intermediate betaine aldehyde. The reaction generates products which react with the Choline Probe to generate fluorescence (Ex/Em 535/587 nm, ab65345 Abcam, Cambridge, UK). Acetylcholine was converted to choline by adding acetylcholinesterase to the reaction and then total choline (choline + acetyl choline) was measured. The amount of acetyl choline was calculated by subtracting choline from total choline. Data are shown as percentage of content Ach or choline with respect to untreated control. Data were normalized to 10^5^ cells.

### 4.4. Lipid Peroxidation Assay

Adult hippocampal neurons were seeded in 6-multiwell plates (1 × 10^5^ cells/well) and incubated with with saline buffer (control cells) or 500 µM GPE for seven days. Following incubation, cells were collected and lysed. The samples were added to 2-thiobarbituric acid (TBA) solution and incubated at 95 °C for 60 min, before cooling to room temperature in an ice bath for 10 min. The malondialdehyde (MDA)-TBA adduct was read by a fluorescence plate reader (Ex/Em 532/553 nm, Ab118970, Abcam, Cambridge, UK). Data are expressed as percentage with respect to untreated cells. Data were normalized to 10^5^ cells.

### 4.5. Membrane Fluidity Assay

Adult hippocampal neurons were seeded in 6-multiwell plates (1 × 10^5^ cells/well) and incubated with with saline buffer (control cells) or 5, 50, or 500 µM GPE for seven days. Following incubation, cells were incubated with the lipophilic pyrene probe, which undergoes excimer formation upon spatial interaction with the cellular membrane (ab189819, Abcam, Cambridge, UK). By measuring the ratio of monomer (Em max 370 nm) to excimer (Em 470 nm) fluorescence, a quantitative monitoring of the membrane fluidity was attained. Data are expressed as ratio between pyrene excimer and monomer. Data were normalized to 10^5^ cells.

### 4.6. Measurement of Autophagic Induction

Adult hippocampal neurons were seeded in 6-multiwell plates (1 × 10^5^ cells/well) and incubated with saline buffer (control cells) or 500 µM GPE for seven days. In selected experiments, the mTOR inhibitor everolimus (50 nM) was used as positive control of autophagy induction [23,24].

Following incubation, cells were collected and lysed for 60 min at 4 °C in RIPA buffer (9.1 mM NaH_2_PO_4_, 1.7 mM Na_2_HPO_4_, 150 mM NaCl, pH 7.4, 0.5% sodium deoxycholate, 1% Nonidet P-40, 0.1% SDS, and a protease-inhibitor cocktail). Cell extracts (40 μg of protein) were diluted in Laemmli sample solution, resolved using SDS-PAGE (8.5%), transferred to PVDF membranes. Then, samples were incubated overnight at 4 °C using the primary antibodies specific for the autophagic markers, i.e., the anti-microtubule-associated protein light chain-3 (LC3B, ab51520, Abcam) and anti-p62 [22]. An antibody specific for -actin (Sigma Aldrich, Milan, Italy, 1:5000) was used as the loading control. The specific peroxidase-conjugated secondary antibodies were then applied and detected using a chemiluminescent substrate (ECL, Perkin Elmer, Milan, Italy). Densitometric analysis of the immunoreactive bands was performed using Image J Software.

### 4.7. Cellular Model of Aging and Quantification of DNA Damage

To establish the aging model, adult hippocampal neurons were maintained in culture for 21 DIV (days in vitro) [25,43]. Cells were cultured in the presence of PBS or GPE (5, 50, and 500 µM). Following incubation, samples were collected and the levels of Double Stand Break (DSB) and phospho-ATM were quantified as parameters of DNA damage by cytofluorimetric analysis (Muse^TM^) [1,44,45]. Data were expressed as percentage with respect to total cell population.

### 4.8. Cell Viability Assay in Aged Cells

Adult hippocampal neurons were maintained in culture for 21 DIV (5 × 10^3^ cells/well in parallel) in the presence of PBS or GPE at different concentrations (5, 50, or 500 µM). After incubation, cellular viability was measured by MTS assay [22,44,45]. Data related to GPE effects were expressed as percentage with respect to total cell population, set to 100%.

### 4.9. Statistical Analysis

Data analysis was performed using the *t*-test and one-way analysis of variance (ANOVA) with Bonferroni’s corrected *t*-tests for post-hoc pair-wise comparisons. *p* < 0.05 was considered statistically significant [1].

## 5. Conclusions

In human hippocampal neurons, GPE was proven to: (i) increase the content of phospholipids (PC and PE) and Ach; (ii) promote the proper membrane function, through a reduction of lipid peroxidation and an enhancement of membrane fluidity; (iii) induce autophagy; (iv) exert cytoprotective effects in aged cells. Overall, these results confirm the beneficial effects of GPE supplementation and suggest the compound as a promising agent to preserve hippocampal neurons and virtually memory performances.

## Abbreviations

Ach	Acetylcholine
DSB	Double Strand Break
GPE	alpha-glycerylphosphorylethanolamine
NSC	Neural Stem Cell
PE	Phosphatidylethanolamine
PC	Phosphatidylcholine
PL	Phospholipid
PUFA	Polyunsaturated n-3 Fatty Acids
